# Association Between Chronic Kidney Disease and Subclinical Hypothyroidism Categorized by Hypertension Status

**DOI:** 10.3390/diseases14080283

**Published:** 2026-08-06

**Authors:** Yuji Shimizu, Asuka Oyama, Yuko Noguchi, Mutsumi Matsuu-Matsuyama, Koichiro Hamada, Shin-Ya Kawashiri, Hirotomo Yamanashi, Seiko Nakamichi, Yasuhiro Nagata, Takahiro Maeda, Naomi Hayashida

**Affiliations:** 1 Department of General Medicine, Nagasaki University Graduate School of Biomedical Sciences, Nagasaki 852-8501, Japan; hmd516@sky.plala.or.jp (K.H.); yamanashi@nagasaki-u.ac.jp (H.Y.); seiko-n@nagasaki-u.ac.jp (S.N.); tmaeda@nagasaki-u.ac.jp (T.M.); 2 Epidemiology Section, Division of Public Health, Osaka Institute of Public Health, Osaka 537-0025, Japan; ooyama@iph.osaka.jp; 3 Department of Community Medicine, Nagasaki University Graduate School of Biomedical Sciences, Nagasaki 852-8523, Japan; y-noguti@nagasaki-u.ac.jp (Y.N.); shin-ya@nagasaki-u.ac.jp (S.-Y.K.); ynagata1961@nagasaki-u.ac.jp (Y.N.); 4 Department of Health Society and Statistics, Atomic Bomb Disease Institute, Nagasaki University, Nagasaki 852-8523, Japan; mutsumi@nagasaki-u.ac.jp (M.M.-M.); naomin@nagasaki-u.ac.jp (N.H.); 5 Leading Medical Research Core Unit, Nagasaki University Graduate School of Biomedical Sciences, Nagasaki 852-8523, Japan; 6 Nagasaki University Health Center, Nagasaki 852-8521, Japan

**Keywords:** chronic kidney disease, subclinical hypothyroidism, hypertension, latent damage, endothelial repair, thyroid hormone, deiodination

## Abstract

Background/Objectives: Subclinical hypothyroidism (SCH) has been reported to be associated with chronic kidney disease (CKD). Anti–thyroid peroxidase antibody (TPO-Ab) positivity, a known cause of autoimmune thyroid disease, has been reported to be positively associated with SCH with hypertension, but not with SCH without hypertension. Therefore, hypertension status might indicate latent thyroid damage among individuals with SCH. This distinction suggests that categorizing SCH by hypertension status could be useful for evaluating its association with CKD. Methods: A cross-sectional study of 1479 Japanese aged 40–69 years, all of whom had thyroid hormone levels (free triiodothyronine and free thyroxine) within the normal range, was conducted to evaluate the association between CKD and SCH categorized by hypertension status. Results: No significant association between SCH without hypertension and CKD was observed. However, a significant positive association between SCH with hypertension and CKD was identified. The potential confounders-adjusted odds ratios (ORs) and 95% confidence intervals (CIs) were 0.96 (0.39, 2.38) for SCH without hypertension and 2.76 (1.37, 5.59) for SCH with hypertension. Conclusions: Although further investigation is warranted, categorizing SCH by hypertension status may be useful for understanding the association between SCH and CKD. These findings may help clarify the biological significance of SCH in the development of CKD.

## 1. Introduction

Deiodination, the process converting thyroxine (T4) to triiodothyronine (T3) [1], reflects thyroid hormone activity, as T3 is the biologically active form while T4 acts as a prohormone [2,3]. Consequently, the free T3 to free T4 ratio (free T3/free T4) serves as a marker of deiodination [4,5] and indicates thyroid hormone activity. The enzymes responsible for this process are present in the liver and kidneys [6,7]. Thus, impaired enzyme activity, potentially driven by renal dysfunction, may be inversely associated with both deiodination and chronic kidney disease (CKD) [8]. Previous studies support this, showing that a lower free T3 /free T4 ratio is associated with CKD [9], which suggests that renal impairment is linked to reduced thyroid hormone activity [10,11]. Given that subclinical hypothyroidism (SCH) is characterized by reduced thyroid hormone activity [12,13], the previously reported positive association between SCH and CKD [14,15,16] aligns with this concept. However, the same study paradoxically reported a positive association between the free T3/free T4 ratio and SCH [14]. Although elucidating this contradiction could yield crucial insights into CKD prevention, no research to date has successfully explained it.

An experimental study in rats found that T3 attenuated the progression of renal injury [17]. Previous studies have demonstrated a strong positive association between serum free T3 levels and renal function in older individuals from the general population [18,19]. Endothelial repair may be an effective strategy for preventing CKD [20,21]. CD34-positive cells are recognized as progenitor cells that contribute to endothelial repair [22,23]. Therefore, the number of circulating CD34-positive cells is considered an indicator of endothelial repair activity [24]. Thyroid hormones have been shown to increase the number of circulating progenitor cells [25,26]. Because T3 is the biologically active form of thyroid hormone, whereas T4 serves primarily as a prohormone, endothelial injury may increase the demand for thyroid hormone by stimulating endothelial repair processes. This increased demand could lead to elevated serum thyroid-stimulating hormone (TSH) levels and eventually contribute to the development of SCH.

Although thyroid hormone production increases, circulating levels of free T3 and free T4 may remain within the normal range because of increased peripheral consumption. These studies indicate that, even in the absence of direct thyroid gland injury, SCH may occur; however, preserved active endothelial repair may limit the extent to which SCH is associated with CKD.

Conversely, anti-thyroid peroxidase antibody (TPO-Ab)—the principal driver of autoimmune thyroiditis [27]—has been shown to elevate TSH levels even in euthyroid individuals with normal free T3 and free T4 ranges [28,29,30]. Because a positive TPO-Ab status uniquely correlates with hypertensive SCH rather than normotensive SCH [31], phenotypic classification of SCH according to blood pressure status may facilitate the differentiation of intrinsic thyroid pathology from generalized subclinical conditions. As this type of SCH reflects a state of thyroid hormone deficiency, a stronger association with CKD may be observed.

However, no study has evaluated the association between SCH categorized by hypertension status and CKD. We therefore hypothesized that, although no significant association would be observed between SCH without hypertension and CKD, a significant association would be observed between SCH with hypertension and CKD.

Furthermore, the development of carotid intima-media thickness (CIMT) depends on angiogenesis of the vasa vasorum [32], whereas angiogenesis may contribute to the prevention of hypertension through a reduction in peripheral vascular resistance [33,34]. Consequently, progression of atherosclerosis as measured by CIMT may, at least in part, represent a process that also counteracts the development of hypertension [35]. If SCH has a favorable effect on active endothelial repair, which contributes to atherosclerosis while mitigating hypertension, the positive association between atherosclerosis and hypertension should be evident only in individuals without SCH. To evaluate these hypotheses, a cross-sectional study of Japanese individuals aged 40 to 69 years who participated in an annual health check-up was conducted. All participants had thyroid hormone levels (free triiodothyronine and free thyroxine) within the normal range.

## 2. Materials and Methods

Details of the present surveys, including the assessment of thyroid function and CIMT, have been reported previously [14,30,31,35].

### 2.1. Study Population

A total of 1582 Japanese residents aged 40–74 years who participated in a community-based annual health checkup in Saza Town, western Japan, in 2014 were initially considered for the study. For the purpose of eliminating confounding biases linked to thyroid status, we omitted individuals with previous or current thyroid treatment (*n* = 25), as well as those who lacked complete records for TSH, free T3, or free T4 (*n* = 16). Additionally, subjects whose free T3 or free T4 levels deviated from the standard reference ranges—specifically 2.1–4.1 pg/mL for free T3 and 1.0–1.7 ng/dL for free T4—were also excluded (*n* = 60). In addition, participants with missing information on blood pressure (*n* = 1) or serum creatinine (*n* = 1) were also excluded. Consequently, 1479 participants (mean age 58.4 years, standard deviation [SD] 8.3 years; range 40–69 years) were included in the final analysis.

### 2.2. Data Collection and Laboratory Measurements

Standardized questionnaires administered by trained interviewers were utilized to gather clinical data. Following an overnight fast, blood samples were obtained from all participants. For blood pressure assessment, individuals remained seated for a minimum of five minutes before trained personnel recorded measurements from the right arm using an automated sphygmomanometer (HEM-907; Omron, Kyoto, Japan). Hypertension was identified by a systolic blood pressure of 140 mmHg or higher, a diastolic blood pressure of 90 mmHg or higher, or the active use of antihypertensive therapies [36]. Chemiluminescent immunoassays conducted at LSI Medience Corporation (Tokyo, Japan) were used to quantify serum concentrations of TSH, free T3, and free T4. The reference intervals—specifically 2.1–4.1 pg/mL for free T3, 1.0–1.7 ng/dL for free T4, and 0.39–4.01 μIU/mL for TSH—aligned with the manufacturer’s specifications for this assay system [37]. Serum TSH levels surpassing 4.01 μIU/mL were classified as SCH.SRL, Inc. (Tokyo, Japan) performed the serum creatinine measurements using routine laboratory procedures. The glomerular filtration rate (GFR) was calculated via the equation developed by the Japanese Chronic Kidney Disease Initiative working group [38]. We defined CKD as an estimated GFR below 60 mL/min/1.73 m^2^ [39].

CIMT was evaluated by ultrasonography of both common carotid arteries using a LOGIQ Book XP system equipped with a 10 MHz linear transducer (GE Healthcare, Milwaukee, WI, USA). Examinations were performed by an experienced vascular technician. Previous studies, including the Carotid Intima Media Thickness (IMT) and IMT-Progression as Predictors of Vascular Events in a High Risk European Population study (IMPROVE study), have suggested that indices incorporating maximum CIMT values provide superior prediction of cardiovascular risk compared with mean CIMT [40,41,42]. Accordingly, the maximum CIMT values of the left and right common carotid arteries were obtained using semi-automated edge-detection software (Intimascope; MediaCross, Tokyo, Japan), following a standardized protocol [43]. This software is designed to improve measurement precision and reduce observer-dependent variability. Atherosclerosis was defined as CIMT ≥ 1.1 mm, consistent with prior studies [44].

A thyroid cyst was defined as a lesion with a maximum diameter ≥ 2.0 mm without any solid component, based on previously established criteria [44,45].

### 2.3. Statistical Analysis

The clinical characteristics of the study population according to SCH status with and without hypertension are presented as mean ± 1 SD for continuous variables, except for TSH, and as percentages for the prevalence of male sex, atherosclerosis, and thyroid cysts. Because TSH values showed a skewed distribution, logarithmic transformation was performed, and the median [interquartile range] is presented.

Logistic regression models were used to calculate odds ratios (ORs) and 95% confidence intervals (CIs) to examine the associations between SCH and CKD, between SCH without hypertension and CKD, and between SCH with hypertension and CKD.

The SCH status–specific association between atherosclerosis and hypertension, as well as the effect of SCH status on the association between atherosclerosis and hypertension, was also evaluated using logistic regression models.

Three adjusted models were used to assess these associations. The first model was adjusted for sex and age only (Model 1). Because SCH status, rather than thyroid hormone levels per se, may determine these associations, the second model was further adjusted for free T3 (Model 2).

CIMT, a marker of atherosclerosis [46], has been reported to be associated with CKD [47,48]. The absence of thyroid cysts has been reported to indicate latent thyroid damage [45]. Thyroid cysts also influence the association between hypertension and atherosclerosis [44].

Therefore, in Model 3, in addition to the variables included in Model 2, atherosclerosis and thyroid cyst were included as confounders, except in analyses evaluating the association between atherosclerosis and hypertension.

Model 3, as presented in the study, was adjusted to include additional confounders (atherosclerosis and thyroid cysts), while using the Hosmer–Lemeshow test to validate the logistic regression models.

In addition, data on thyroid peroxidase antibody (TPO-Ab) levels were available for 1222 individuals in the study population. Based on previous reports, TPO-Ab levels <16 IU/mL were considered within the normal range [37]. Accordingly, TPO-Ab positivity was defined as a level ≥16 IU/mL in the present study [31]. In an additional analysis, three multivariable-adjusted models were used to evaluate the association between TPO-Ab positivity andSCH without hypertension, as well as the association between TPO-Ab positivity and SCH with hypertension.

Data analysis was conducted using SAS version 9.4, with statistical significance set at *p* < 0.05 for main effects and *p* < 0.20 for interactions, consistent with established literature [47,49]. For more details, consult the provided statistical analysis description.

## 3. Results

Among the study population, 81 individuals had SCH; 44 had SCH with hypertension, 37 had SCH without hypertension, and 221 had CKD.

### 3.1. Clinical Characteristics of the Study Population

Table 1 presents the clinical characteristics of the study population.

#### 3.1.1. Characteristics by SCH Status Without Hypertension

Compared with non-SCH individuals without hypertension, those with SCH without hypertension had significantly higher TSH levels, significantly lower free T4 levels, and a higher prevalence of atherosclerosis, although the latter association was not statistically significant.

#### 3.1.2. Characteristics by SCH Status with Hypertension

Compared with non-SCH individuals with hypertension, those with SCH with hypertension were significantly older, had significantly higher TSH levels, and had significantly lower free T4 levels.

Even if the sample sizes for SCH with hypertension and SCH without hypertension are small, TSH levels are significantly higher and free T4 levels are significantly lower in SCH without hypertension compared to non-SCH without hypertension, as well as in SCH with hypertension compared to non-SCH with hypertension. Therefore, it is suggested that the characteristics of SCH with hypertension and SCH without hypertension do not significantly interfere with each other.

### 3.2. Association Between CKD and SCH

SCH showed a significant positive association with CKD in all adjusted models (Table 2). The adjusted ORs (95% CIs) were 1.87 (1.09, 3.23) in Model 1, 1.79 (1.03, 3.10) in Model 2, and 1.79 (1.03, 3.09) in Model 3.

### 3.3. Association Between CKD and SCH Without Hypertension

No significant association between CKD and SCH without hypertension was observed (Table 3). The sex- and age-adjusted OR (95% CI) for CKD in participants with SCH without hypertension was 1.02 (0.41, 2.50) (Model 1). The ORs (95% CIs) were similar in Model 2 and Model 3, with corresponding values of 0.98 (0.40, 2.41) and 0.96 (0.39, 2.38), respectively.

### 3.4. Association Between CKD and SCH with Hypertension

A significant positive association between CKD and SCH with hypertension was observed (Table 4). The adjusted ORs (95% CIs) were 2.87 (1.42, 5.79) in Model 1, 2.73 (1.35, 5.52) in Model 2, and 2.76 (1.37, 5.59) in Model 3.

As a sensitivity analysis, renal dysfunction was alternatively defined as a GFR < 90 mL/min/1.73 m^2^ and as a GFR < 45 mL/min/1.73 m^2^. The observed associations were essentially unchanged. Using the age- and sex-adjusted model and defining renal dysfunction as a GFR < 90 mL/min/1.73 m^2^ [50], with non-SCH participants without hypertension as the reference group (*n* = 1435; cases = 1324), the adjusted OR (95% CI) for SCH without hypertension (*n* = 44; cases = 39) was 0.75 (0.30, 1.98). Similarly, with non-SCH participants with hypertension as the reference group (*n* = 1442; cases = 1327), the adjusted OR (95% CI) for SCH with hypertension (*n* = 37; cases = 36) was 2.44 (0.33, 18.14). When renal dysfunction was defined as a GFR < 45 mL/min/1.73 m^2^ [50], the adjusted OR for SCH without hypertension could not be estimated because no cases of renal dysfunction were observed in this subgroup (non-SCH without hypertension: *n* = 1435, cases = 19; SCH without hypertension: *n* = 44, cases = 0). In contrast, with non-SCH participants with hypertension as the reference group (*n* = 1442; cases = 15), the adjusted OR (95% CI) for SCH with hypertension (*n* = 37; cases = 4) was 8.88 (2.75, 28.74). Overall, despite the limited number of SCH cases, the direction of the associations remained largely consistent across different definitions of renal dysfunction, supporting the robustness of the main findings.

### 3.5. Association Between TPO-Ab Positivity and SCH Without and with Hypertension

Table 5 presents the associations of TPO-Ab positivity with SCH without hypertension and SCH with hypertension. SCH without hypertension was not significantly associated with TPO-Ab positivity. In contrast, SCH with hypertension was significantly and positively associated with TPO-Ab positivity. These findings remained essentially unchanged across all three adjusted models.

### 3.6. Association Between Hypertension and Atherosclerosis by SCH Status

Table 6 presents the SCH status–specific association between hypertension and atherosclerosis. In all adjusted models, significant positive associations were observed between hypertension and atherosclerosis among individuals without SCH. Among individuals with SCH, the associations did not reach statistical significance; however, an inverse trend between hypertension and atherosclerosis was observed in all adjusted models. SCH status showed a significant interaction effect on the association between hypertension and atherosclerosis in all adjusted models.

## 4. Discussion

The major finding of the present study was that, although a significant positive association was observed between SCH with hypertension and CKD, no significant association was observed between SCH without hypertension and CKD.

A case–control study including 200 individuals with CKD and 200 without CKD reported a significant association between SCH and CKD [51]. In a nationwide population-based cross-sectional study of 3257 individuals, the prevalence of CKD increased stepwise with decreasing thyroid function, from 1.5% in subclinical hyperthyroidism to 6.6% in euthyroidism and 12.6% in subclinical hypothyroidism (*p* for trend < 0.001) [52]. These findings are consistent with our results, which showed a significant positive association between SCH and CKD.

In the present study, we further demonstrated that a significant positive association was observed between SCH with hypertension and CKD, but not between SCH without hypertension and CKD. To our knowledge, this is the first study to report the association between SCH and CKD with a specific focus on hypertension status.

Variations in the pathophysiology of SCH, depending on the presence or absence of hypertension, might underlie these observed associations. The previous study revealed a positive association between SCH and the free T3/free T4 ratio [14]. This finding suggests that individuals with SCH may have higher thyroid hormone activity than those without SCH. The same study also reported a positive association between SCH and CKD [14]. These findings indicate that physiological conditions that stimulate deiodination may also induce SCH, whereas deiodination itself may exert a protective effect against CKD by activating endothelial repair. Since endothelial injury increases the demand for endothelial repair, it may also increase the demand for biologically active thyroid hormone (T3). This process may accelerate deiodination of T4 to T3, reflecting increased thyroid hormone utilization. Consequently, the resulting increase in thyroid hormone demand may lead to elevated TSH levels and eventually manifest as SCH.

There appear to be two main pathophysiological mechanisms underlying the development of SCH: increased thyroid hormone demand and latent thyroid gland damage. Because thyroid hormone is expected to have a beneficial effect in preventing CKD [14,17], the former mechanism may contribute to CKD prevention, whereas the latter mechanism may not prevent CKD because of reduced thyroid hormone effectiveness. In the present study, SCH accompanied by hypertension showed a significant positive association with CKD, whereas SCH without hypertension did not. These findings suggest that hypertension may serve as a useful clinical indicator for distinguishing between these two forms of SCH.

In our additional analysis, TPO-Ab positivity, a known indicator of autoimmune thyroiditis, was significantly associated with SCH with hypertension, whereas no such association was observed for SCH without hypertension, which is consistent with our previous report [31]. Therefore, SCH accompanied by hypertension may reflect underlying latent thyroid damage, while SCH without hypertension may arise through mechanisms other than latent thyroid gland damage.

CKD has been reported to be associated with endothelial dysfunction [20,53], which contributes to the development of atherosclerosis, as evaluated by CIMT [47]. Endothelial cells [54], macrophages [55], and foam cells [56] represent major cellular sources of atherosclerotic lesions. CD34-positive progenitor cells contribute to vascular remodeling, including the development of CIMT, through their differentiation into endothelial progenitor cells [22], macrophages [57], foam cells [58], and megakaryocytes [59]. Because the progression of CIMT is associated with angiogenesis [32], and impaired angiogenesis is a recognized risk factor for hypertension [33,34], these processes appear to be closely linked. Moreover, thyroid hormone has been shown to increase circulating progenitor cells [25,26]. Taken together, these findings suggest that the progression of atherosclerosis, as assessed by CIMT, may reflect compensatory vascular remodeling that helps reduce peripheral vascular resistance and thereby contributes to blood pressure regulation [35]. Accordingly, SCH may influence hypertension through its effects on the process of atherosclerosis development, as reflected by CIMT.

As shown in Table 6, a significant positive association between hypertension and atherosclerosis was observed only among individuals without SCH. Among those with SCH, although the statistical power was limited and the association did not reach statistical significance, an inverse trend between hypertension and atherosclerosis was observed. These findings support the hypothesis that SCH may modify the association between hypertension and atherosclerosis progression.

Because atherosclerosis development involves endothelial repair [35], which is associated with increased thyroid hormone demand [25,26], SCH without hypertension may reflect a state induced by increased demand related to endothelial repair. In contrast, when SCH is induced by latent thyroid gland damage, thyroid hormone activity may be insufficient to prevent hypertension. Therefore, SCH with hypertension may reflect SCH associated with latent thyroid gland damage.

T3 has been reported to reduce hypertension-related endothelial dysfunction [60]. TPO-Ab is a well-established autoimmune marker of autoimmune thyroid disease [28,29,30]. A previous study reported that TPO-Ab positivity was positively associated with SCH with hypertension, but not with SCH without hypertension [31]. This evidence partially supports the proposed mechanisms, suggesting that latent thyroid gland damage related to TPO-Ab positivity may be associated with SCH with hypertension, but not with SCH without hypertension.

The present study has several clinical implications. In addition to increased thyroid hormone demand, which may contribute to CKD prevention, latent thyroid gland damage may represent an important pathophysiological mechanism underlying SCH. Hypertension status may serve as a useful clinical indicator for categorizing SCH according to its underlying pathophysiology. When developing treatment strategies for SCH, hypertension status may provide clinically relevant information.

Several potential limitations of the present study warrant consideration. Although latent thyroid gland damage may underlie the pathophysiology of SCH with hypertension, we were unable to evaluate the direct association between latent thyroid gland damage and SCH with hypertension because no established method is currently available to assess subclinical thyroid gland damage. Further investigations incorporating artificial intelligence–based analysis of thyroid ultrasound images may be warranted. Although the number of CKD cases was small among participants with SCH and hypertension (*n* = 37), the association with CKD was stronger for SCH with hypertension than for SCH overall. In other words, despite restricting the analysis to a more specific subgroup, the magnitude of the association increased rather than decreased. In addition, sensitivity analyses using alternative GFR cutoffs (<90 and <45 mL/min/1.73 m^2^) showed essentially similar results. However, given the limited sample size, these findings should be interpreted with caution.

Because only two individuals with SCH and atherosclerosis had hypertension, the statistical power was insufficient to detect a significant association between hypertension and atherosclerosis in this subgroup. Therefore, further investigation with a larger study population is required. Because this study was cross-sectional in design, causal relationships cannot be established.

## 5. Conclusions

Although a significant positive association was observed between SCH with hypertension and CKD, no significant association was observed between SCH without hypertension and CKD. These findings may contribute to the development of improved approaches for evaluating thyroid function and to a better understanding of the pathophysiology of SCH. In addition, these findings may inform strategies for CKD prevention.

## Figures and Tables

**Table 1:** Characteristics of the study population.

	SCH Without Hypertension		*p*-Value	SCH with Hypertension		*p*-Value
	(−)	(+)		(−)	(+)	
No. of participants	1435	44		1422	37	
Men, %	34.4	36.4	0.783	34.3	40.5	0.427
Age, years	58.4 ± 8.3	56.4 ± 9.3	0.112	58.3 ± 8.3	62.4 ± 6.7	0.003
TSH, μIU/mL	1.5 [1.1, 2.2] *^1^	4.8 [4.3, 5.38] *^1^	<0.001 *^2^	1.5 [1.1, 2.2] *^1^	5.5 [4.5, 6.5] *^1^	<0.001 *^2^
Free T3, pg/mL	3.2 ± 0.3	3.1 ± 0.3	0.291	3.2 ± 0.3	3.1 ± 0.3	0.170
Free T4, ng/dL	1.3 ± 0.2	1.2 ± 0.2	0.021	1.3 ± 0.2	1.2 ± 0.2	0.004
free T3/ free T4 ratio	2.57 ± 0.34	2.65 ± 0.35	0.123	2.57 ± 0.35	2.68 ± 0.35	0.066
Atherosclerosis, %	8.2	15.9	0.071	8.5	5.4	0.500
Thyroid cyst, %	31.6	36.4	0.508	31.9	27.0	0.530

Abbreviations: TSH, thyroid-stimulating hormone; T3, triiodothyronine; T4, thyroxine; CKD, chronic kidney disease. The mean ± 1 standard deviation was used to describe age, free T3, free T4, and the free T3/ free T4 ratio. *^1^: These variables are reported as median [25th and 75th percentiles]. *^2^: Statistical *p*-values were evaluated after applying a logarithmic transformation.

**Table 2:** Association between CKD and SCH.

	SCH		*p*-Value
	(−)	(+)	
No. of participants	1398	81	
No. of CKD (%)	201 (14.4)	20 (24.7)	
Model 1	Ref	1.87 (1.09, 3.23)	0.024
Model 2	Ref	1.79 (1.03, 3.10)	0.037
Model 3	Ref	1.79 (1.03, 3.09)	0.038

Abbreviations: Ref, reference; CKD, chronic kidney disease; SCH, subclinical hypothyroidism. Model 1 was adjusted for sex and age. Model 2 was adjusted for sex, age, and free triiodothyronine (T3). Model 3 was adjusted for the variables included in Model 2, with additional adjustment for atherosclerosis and thyroid cysts.

**Table 3:** Association between CKD and SCH without Hypertension.

	SCH Without Hypertension		*p*-Value
	(−)	(+)	
No. of participants	1435	44	
No. of CKD (%)	215 (15.0)	6 (13.6)	
Model 1	Ref	1.02 (0.41, 2.50)	0.971
Model 2	Ref	0.98 (0.40, 2.41)	0.961
Model 3	Ref	0.96 (0.39, 2.38)	0.936

Abbreviations: Ref, reference; CKD, chronic kidney disease; SCH, subclinical hypothyroidism. Model 1 was adjusted for sex and age. Model 2 was adjusted for sex, age, and free triiodothyronine (T3). Model 3 was adjusted for the variables included in Model 2, with additional adjustment for atherosclerosis and thyroid cysts.

**Table 4:** Association between CKD and SCH with Hypertension.

	SCH with Hypertension		*p*-Value
	(−)	(+)	
No. of participants	1442	37	
No. of CKD (%)	207 (14.4)	14 (37.8)	
Model 1	Ref	2.87 (1.42, 5.79)	0.003
Model 2	Ref	2.73 (1.35, 5.52)	0.005
Model 3	Ref	2.76 (1.37, 5.59)	0.005

Abbreviations: Ref, reference; CKD, chronic kidney disease; SCH, subclinical hypothyroidism. Model 1 was adjusted for sex and age. Model 2 was adjusted for sex, age, and free triiodothyronine (T3). Model 3 was adjusted for the variables included in Model 2, with additional adjustment for atherosclerosis and thyroid cysts.

**Table 5:** Association between TPO-Ab positivity and SCH without and with hypertension.

	SCH Without Hypertension		*p*-Value	SCH with Hypertension		*p*-Value
	(−)	(+)		(−)	(+)	
No. of participants	1185	37		1193	29	
No. of TPO-Ab positivity (%)	221 (18.6)	7 (18.9)		217 (18.2)	11 (37.9)	
Model 1	Ref	1.05 (0.45, 2.44)	0.906	Ref	2.64 (1.22, 5.73)	0.014
Model 2	Ref	1.06 (0.46, 2.45)	0.899	Ref	2.67 (1.23, 5.78)	0.013
Model 3	Ref	1.14 (0.49, 2.67)	0.760	Ref	2.61 (1.19, 5.73)	0.017

Abbreviations: Ref, reference; SCH, subclinical hypothyroidism; TPO-Ab, anti-thyroid peroxidase antibody. Model 1 was adjusted for sex and age. Model 2 was adjusted for sex, age, and free triiodothyronine (T3). Model 3 was adjusted for the variables included in Model 2, with additional adjustment for atherosclerosis and thyroid cysts.

**Table 6:** Association between hypertension and atherosclerosis by SCH status.

	SCH						Interaction (*p*-Value)
	(−)			(+)			
	Atherosclerosis		*p*-Value	Atherosclerosis		*p*-Value	
	(−)	(+)		(−)	(+)		
No. of participants	1282	116		72	9		
No. of hypertension (%)	432 (33.7)	63 (54.3)		35 (48.6)	2 (22.2)		
Model 1	Ref	1.55 (1.03, 2.33)	0.037	Ref	0.18 (0.03, 1.04)	0.055	0.094
Model 2	Ref	1.57 (1.04, 2.37)	0.032	Ref	0.19 (0.03, 1.13)	0.068	0.089
Model 3	Ref	1.53 (1.01, 2.31)	0.038	Ref	0.25 (0.04, 1.49)	0.128	0.067

Abbreviations: Ref, reference; SCH, subclinical hypothyroidism. Model 1 was adjusted for sex and age. Model 2 was adjusted for sex, age, and free triiodothyronine (T3). Model 3 was adjusted for the variables included in Model 2, with additional adjustment for thyroid cysts.

## Data Availability

For the studies described in this review, we cannot publicly provide individual data because of participant confidentiality considerations according to the ethical guidelines in Japan. Additionally, obtaining informed consent does not include a provision for publicly sharing the data. Qualified researchers have access to minimal datasets. Please contact the Office of Data Management at ritouken@vc.fctv-net.jp. Information regarding the data request is available at http://www.med.nagasaki-u.ac.jp/cm/ (accessed on 17 June 2026).

## Abbreviations

The following abbreviations are used in this manuscript:

SCH	Subclinical hypothyroidism
CKD	Chronic kidney disease
TPO-Ab	Anti-thyroid peroxidase
CIMT	Carotid intima-media thickness
ORs	Odds ratios
CIs	Confidence intervals
T3	Triiodothyronine
T4	Thyroxine
TSH	Thyroid-stimulating hormone
SD	Standard deviation
GFR	Glomerular filtration rate
